# Quantifying the zoonotic risk profile of European influenza A viruses in swine from 2010 to 2020 inclusive

**DOI:** 10.1128/jvi.00306-25

**Published:** 2025-06-04

**Authors:** Amelia Coggon, Sara Lopes, Gaëlle Simon, Zebulun Arendsee, Kuan-Fu Chen, Chiara Chiapponi, Steve Essen, Helen Everett, Séverine Hervé, David E. Hufnagel, Benjamin Mollett, Ana Moreno, Andrew Pekosz, Gautier Richard, Richard E. Rothman, Kathryn Shaw-Saliba, Kristien Van Reeth, Divya Venkatesh, Ian H. Brown, Tavis K. Anderson, Amy L. Baker, Nicola S. Lewis

**Affiliations:** 1 Royal Veterinary College 4912, London, United Kingdom; 2 Swine Virology Immunology Unit, Ploufragan-Plouzané-Niort Laboratory, French Agency for Food, Environmental and Occupational Health and Safety (ANSES), Ploufragan, France; 3 National Animal Disease Center, Agricultural Research Service, United States Department of Agriculture 57837 , Ames, Iowa, USA; 4 Department of Emergency Medicine, Chang Gung Memorial Hospital, Keelung, Taiwan; 5 College of Intelligent Computing, Chang Gung University 56081, Taoyuan, Taiwan; 6 Istituto Zooprofilattico Sperimentale della Lombardia e dell'Emilia Romagna "Bruno Ubertini", WOAH Reference Laboratory for Swine Influenza, Brescia, Italy; 7 Animal and Plant Health Agency (APHA), Weybridge, United Kingdom; 8 Department of Molecular Microbiology and Immunology, Johns Hopkins Bloomberg School of Public Health, Johns Hopkins University 310946 , Baltimore, Maryland, USA; 9 Department of Emergency Medicine, Johns Hopkins University School of Medicine 1500, Baltimore, Maryland, USA; 10 Laboratory of Virology, Ghent University 26656, Ghent, Belgium; 11 Department of Biology, University of Oxford 98459, Oxford, United Kingdom; 12 The Pirbright Institute, Pirbright, United Kingdom; 13 Worldwide Influenza Centre, London, United Kingdom; Cornell University Baker Institute for Animal Health, Ithaca, New York, USA

**Keywords:** zoonotic influenza A Virus, European swine, antigenic cartography, pre-pandemic risk assessment, genetic diversity, genetic evolution, antigenic evolution

## Abstract

H1 and H3 influenza A viruses (IAVs) circulating in European pigs are markedly distinct from those circulating in other global swine populations. These viruses exhibit significant genetic diversity, further expanded by periodic interspecies transmission of IAVs from humans into pigs, followed by sustained circulation. Several zoonotic IAV infections in humans in Europe have been associated with the 1C lineage of H1 IAVs. Given the predominance of H1 detections in pigs and their zoonotic potential, we quantified antigenic evolution of H1 viruses in European pigs using ferret and pig models and assessed diversity relative to swine IAV vaccine strains. Ferret and swine antisera comparisons revealed no significant differences in antibody responses. Viruses of the 1A.3.3.2 clade exhibited reduced cross-reactivity to human seasonal vaccine strains from 2009. Viruses of the 1B.1.2.2 clade showed no cross-reactivity to the 1978 human seasonal influenza viruses nor to candidate vaccine viruses (CVVs). Clades 1C.2.1 and 1C.2.2 human variant strains had variable cross-reactivity to the tested 1C lineage CVVs, and 1C.2.4 and 1C.2.5 clade viruses exhibited rapid genetic diversification. Many viruses tested were antigenically distant from swine influenza vaccine-representative strains, highlighting the need for updated vaccine formulations. Importantly, age-stratified human serum panels revealed limited population cross-protection to tested viruses, particularly for antigenically heterogenous viruses. These findings quantify the genetic and antigenic diversity of co-circulating IAV lineages and identify specific groups of viruses that may represent a greater risk to animal and public health. These results can be used to inform future pre-pandemic preparedness efforts.

**IMPORTANCE:**

Our data demonstrate the importance of matching swine influenza vaccine seed strains to contemporary circulating viruses and highlight how vaccine mismatch may drive additional antigenic evolution and demonstrate the need for constant monitoring and surveillance of swine viruses for the benefit of both animal and global health. These findings can be used to inform and prioritize future pre-pandemic preparedness efforts.

## INTRODUCTION

Swine influenza A virus (IAV) causes respiratory disease in pigs that is of significant economic concern in Europe. In the United Kingdom alone, approximately 50% of the adult pig population has been exposed to one or more strains of IAV over their lifetime (1). Three predominant lineages of swine IAV H1 viruses (1A, 1B, and 1C) and multiple lineages of H3 from human seasonal IAV, which have been established over several decades, continuously circulate in global pig populations (2). Following the spread of the 2009 H1N1 swine-origin pandemic (pdm09) in humans, the annual introduction of human seasonal H1N1pdm09 virus into pigs has led to over a decade of co-circulation, reassortment, and diversification of the hemagglutinin (HA), neuraminidase (NA), and the other six viral genes in endemic swine IAV lineages (1, 3–9). This diversity has important implications for both pig health and control of IAV using vaccines and poses a challenge for pre-pandemic preparedness for the global public health community.

A coordinated European swine IAV surveillance network identified 23 distinct gene constellations of enzootic 1A lineage (pdm09/1A.3.3.2 clade), H1N1 Eurasian avian-like 1C lineage, H1N2 human seasonal 1B lineage, and human-like H3N2 subtypes in European pigs from 2010 to 2013 (9). Many constellations involved internal gene cassettes from Eurasian avian-like or pdm09 viruses with various NA pairings. These viruses have continued to reassort with an additional 16 new genotypes described by Henritzi et al. (1), many of which were associated with reassortment and the acquisition of pdm09 genes. Novel reassortants have been reported in geographically restricted areas reflecting the co-circulation of pdm09 with regionally enzootic swine IAV, in the Netherlands and Belgium (4), France (3), Denmark (10), and Germany (11). These genetically diverse lineages and reassorted viruses have different phenotypes and antigenic characteristics that complicate control measures and pose a zoonotic and pre-pandemic concern, given that all these viruses maintain human-origin genes.

A strategy to minimize the economic losses associated with disease burden in pigs, which also reduces the risk of IAV transmission from pigs to humans, is the use of vaccines in pig populations. In Europe, most commercial vaccines against IAV in swine are inactivated, multivalent (H1N1, H3N2, and/or H1N2) whole‐virus vaccines with an adjuvant. There is no formal system for recommending swine influenza vaccine strains, and the current diversity in IAVs in pigs complicates control by use of inactivated vaccines (8). Formulating effective vaccines is challenged by the difficulty in updating vaccine seed viruses given the emergence of novel lineages and clades, antigenic drift, the time needed to approve and license veterinary vaccine products, maternal antibody interference, and the lack of adequate mucosal and cell-mediated immune responses (12). Furthermore, contributing to observed evolution and subsequent antigenic diversity, positive selection has been demonstrated at antibody epitopes in swine IAV HA genes and may result from partially effective vaccines (13). Influenza vaccines are primarily used in adult sows to clinically protect the gestating sow and her suckling piglets or, less frequently, during the grow/finish phase of production to decrease IAV disease, lung lesions, and transmission (8, 14–16). Vaccinating piglets may be desired in some clinical situations, but the presence of passive maternal antibodies interferes with the efficacy of inactivated vaccines (17).

The potential for another swine-origin IAV pandemic is reinforced by relatively frequent swine-to-human transmission events. Between February 2020 and September 2021, 38 zoonotic cases of swine IAV H1 and H3 viruses (referred to as “variants”) were reported to the World Health Organization (WHO) (18–20). Seven of these cases occurred in Europe and were caused by diverse H1 viruses of H1N1 and H1N2 subtypes, of different lineages and genome constellations, representing the complexity of currently circulating IAV in pigs in Europe. These zoonotic spillovers were predominantly caused by the Eurasian avian 1C lineage. Two H1N1 1C.2.1 clade viruses were reported in humans in the Netherlands (21) and Germany (22), respectively, and one H1N2 1C.2.4 clade virus was reported in France (23). In 2021, there was also a report of a variant virus from the H1N1 1A.3.3.2 clade in Denmark (24) and finally of a subtyped, but unknown clade, of H1N1 virus detected in Austria.

The risk of a swine-to-human transmission event is likely dependent on animal production systems that could differ in the relative degree of human-pig exposure, type of animal-human interface (e.g., live animal markets, exhibition practices, and large commercial farms), viral ecology, the immune profile of the individual, and virus factors (25). Given the increase in genomic surveillance for IAV, it is now possible to address which viruses are circulating in pigs and how this genetic diversity influences antigenic profiles and determine whether there are adequate pandemic preparedness strategies. Currently, most swine IAVs significantly differ from the current H1 and H3 components of human IAV vaccines (2). Few of the genetic clades detected in swine globally include isolates that are genetically matched to a WHO pre-pandemic preparedness candidate vaccine virus (CVV), and the available CVVs might not provide protection given observed genetic and antigenic differences in circulating swine viruses.

Given the gaps in understanding the impact of swine IAV diversity in Europe on pre-pandemic preparedness, we characterized the genetic and antigenic evolution of the major swine IAV lineages in Europe and evaluated antigenic evolution relative to CVVs and swine influenza vaccine-representative strains. Finally, we tested select viruses against human serology panels to infer population immunity against European IAV in swine. Our data demonstrate that swine IAVs in Europe continue to diversify, and we suggest that a comprehensive understanding of the current evolutionary diversity is required to provide assessments on whether current vaccine strains match circulating diversity and how this diversity impacts pre-pandemic risk.

## MATERIALS AND METHODS

### Viruses and sequence data

Viruses from circulating H1 and H3 strains in European pig populations from 2010 to 2020 inclusive were shared by European collaborators within the joint World Organisation for Animal Health and the Food and Agricultural Organization (WOAH-FAO) network of expertise on animal influenza (OFFLU) swine influenza virus technical activity group. Further sequence data from the Global Initiative on Sharing All Influenza Data EpiFlu Database (www.gisaid.org) and GenBank (https://www.ncbi.nlm.nih.gov/genomes/FLU/Database/nph-select.cgi) were added to the data set along with human variant sequence data. Geographical maps with the country of sampling and genetic information were generated in MicroReact (26). Viruses were propagated in Madin-Darby canine kidney cells or embryonated hen eggs, 9–11 days post-incubation. Cell culture supernatant or allantoic fluid was harvested and clarified by centrifugation. Viruses were then ultracentrifuged, and pellets were resuspended overnight at 4°C in sterile phosphate buffered saline at pH 7.4 and stored at −70°C.

### Phylogenetic analysis and strain selection

Sequence alignments were generated for H1 and H3 segments using default settings in MAFFT v7.490 (27). Maximum likelihood (ML) phylogenetic trees were inferred for the H1 segments using IQ-Tree v2.2.6 (28). Phylogenetic trees were visualized in RStudio 4.3.0 using the ggtree package (29). The lineage of the H1 and H3 viruses was assessed using the swine H1 Clade Classification system (30) and the octoFLU pipeline (31). For each of the identified H1 and H3 contemporary lineages, an HA1 protein consensus sequence was generated. Tabular comparisons of amino acid similarity between current CVVs or human seasonal vaccines and representative swine strains were generated using flutile v0.13.0 (https://github.com/flu-crew/flutile). Swine viruses that represent H1 clades that circulated within European pig populations within this study period were selected for further antigenic characterization.

### Antiserum production

Swine antisera to H1 viruses were generated as previously described in Chastagner et al. (32) at the Animal and Plant Health Agency, UK. Additional European-specific antisera raised against French strains collected between 1982 and 2015 were provided by the French Agency for Food, Environmental and Occupational Health & Safety (ANSES) (Data S1). Briefly, pathogen-free pigs (9 weeks old) were inoculated intranasally with live virus (4 mL 10^7^–10^8^ ELD_50_). Three weeks later, the animals were inoculated intramuscularly with the same dose of virus diluted in the adjuvant Montanide ISA 206 (3 mL). Two weeks later, monovalent swine antisera were collected.

Ferret antisera produced against CVV strains (A/Ohio/09/2015, A/Michigan/383/2018) were provided by the Virology, Surveillance and Diagnosis Branch, Influenza Division, Centers for Disease Control and Prevention (CDC), Atlanta, Georgia, USA. Ferret antisera produced against human seasonal vaccine strains, variant strains, and CVVs (A/California/4/2009, A/Brisbane/02/2018, A/Brazil/11/1978, A/Pavia/65/2016, A/Michigan/45/2015, and A/Netherlands/3315/2016) were provided by the Worldwide Influenza Centre at the Francis Crick Institute. Ferret post-infection monovalent antisera produced against European viruses (A/Swine/Germany/AR2749/2015, A/Swine/Netherlands/AR647/2015, A/Swine/Netherlands/AR647/2015, and A/Swine/Germany/AR2749/2015) were provided from the National Reference Laboratory for Avian Influenza, FLI, Germany. The remaining ferret post-infection monovalent antisera were generated at the US Department of Agriculture (USDA) National Animal Disease Center, Ames, Iowa, USA, by live exposure inoculation of pathogen-free ferrets. Ferrets were exsanguinated, and sera were collected 2–3 weeks post-infection (Data S2).

### HI assays

Hemagglutination inhibition (HI) assays were generated according to standard methods previously described (33). Swine and human antisera were treated with receptor-destroying enzyme and heat-inactivated at 56°C for 30 min and adsorbed with 50% turkey red blood cells (RBC) to remove nonspecific inhibitors of hemagglutinin. Ferret antisera were heat-inactivated at 56°C for 30 min, treated with 20% kaolin, and adsorbed with 0.75% guinea RBC to remove nonspecific inhibitors of hemagglutinin. Serial twofold dilutions starting at 1:10 were tested for their ability to inhibit the agglutination of 0.5% turkey RBC with 4–8 hemagglutinating units of virus. HI assays were performed according to standard techniques as described in the World Organization for Animal Health manual of diagnostic tests using ferret and human antisera with guinea pig RBC and turkey RBC, respectively, to measure as described in Anderson et al. (2).

### Antigenic cartography, vaccine antigen assessment, and antibody landscaping

Antigenic cartography was performed using HI assay data with both swine and ferret antisera to generate two- and three-dimensional (2D and 3D) antigenic maps (one using ferret and one using swine antisera) to visualize and quantify the antigenic interrelationships between the H1 influenza A strains as previously described (34, 35). Antigenic maps were created using RStudio v4.3.0 and Racmacs v1.2.9. The strains in the antigenic maps were colored by genetic lineage and clade. One antigenic unit (AU) is equivalent to a two-fold difference in titer in the HI assay.

Antibody landscapes were generated using RStudio 1.4.1106 as previously described (36, 37). The HI titers were plotted in the third dimension as a smooth landscape over the ferret 2D antigenic map. As the minimum dilution used in the HI assay was 1:10, undetectable titers were given a set value of <10. The *X* and *Y* axes of the antibody landscape represent the antigenic distance between the different antigens, while the *Z* axis (height of the landscape) represents the antibody titers (geometric mean value of each respective virus strain). The antibody landscapes visually represent the breadth of the antibody responses of each host (or hosts) to infection. The antibody landscapes were statistically compared using a *t*-test in Excel.

To assess vaccine antigen match in pigs, the antigenic distance between sera raised in ferrets against swine vaccine-representative strains and the European test viruses was inferred using antigenic cartography. The antigenic distance between sera and test strains was visualized by plotting in numbers. Three AU difference between CVV sera and test antigen is considered antigenically different enough to trigger an update to the human influenza vaccine (38).

### Human serum cohorts

Human cohort sera representing different geographic and demographic populations (39, 40) were tested against contemporary European swine H1 and H3 IAV, human seasonal virus reference strains, and CVVs to assess human seasonal influenza-derived antibody recognition of swine strains and to identify IAV diversity that would likely not be covered either by human seasonal vaccine strain immunity or by putative pandemic preparedness CVV strain responses. Two cohorts of human sera were tested against the selected swine H1 and H3 viruses. A convalescent cohort was composed of influenza-infected individuals (*n* = 10) from Taiwan and Hong Kong (season 2016–2017), with sera collected at 28 days post-infection. Sera from this cohort were previously tested and demonstrated HI titers against the H1 vaccine strain A/Michigan/45/2015 (41) and the H3N2 vaccine strain A/Hong Kong/4801/2014 (42). A post-vaccination cohort consisted of subjects from Johns Hopkins Hospital (Baltimore, MD, USA) vaccinated in the autumn of 2017 with a quadrivalent influenza vaccine. Age and gender metadata were provided, so female and male samples were grouped and stratified by decade of age: 1940–1950s, 1950–1960s, 1960–1970,1970-1980s, and 1980–1990s. Five subjects per age group per gender were randomly selected for the final cohort panel (*n* = 50). Patients were enrolled at the Johns Hopkins Medical Institute Department of Emergency Medicine or on inpatient floors. Log_2_ transformed geometric mean titers (GMT) of HI responses were calculated. The data were visualized using boxplots generated with ggplot in RStudio (version 4.3.0).

## RESULTS

### Genomic epidemiology of HA genes of IAV in European pigs

**Fig 1:**
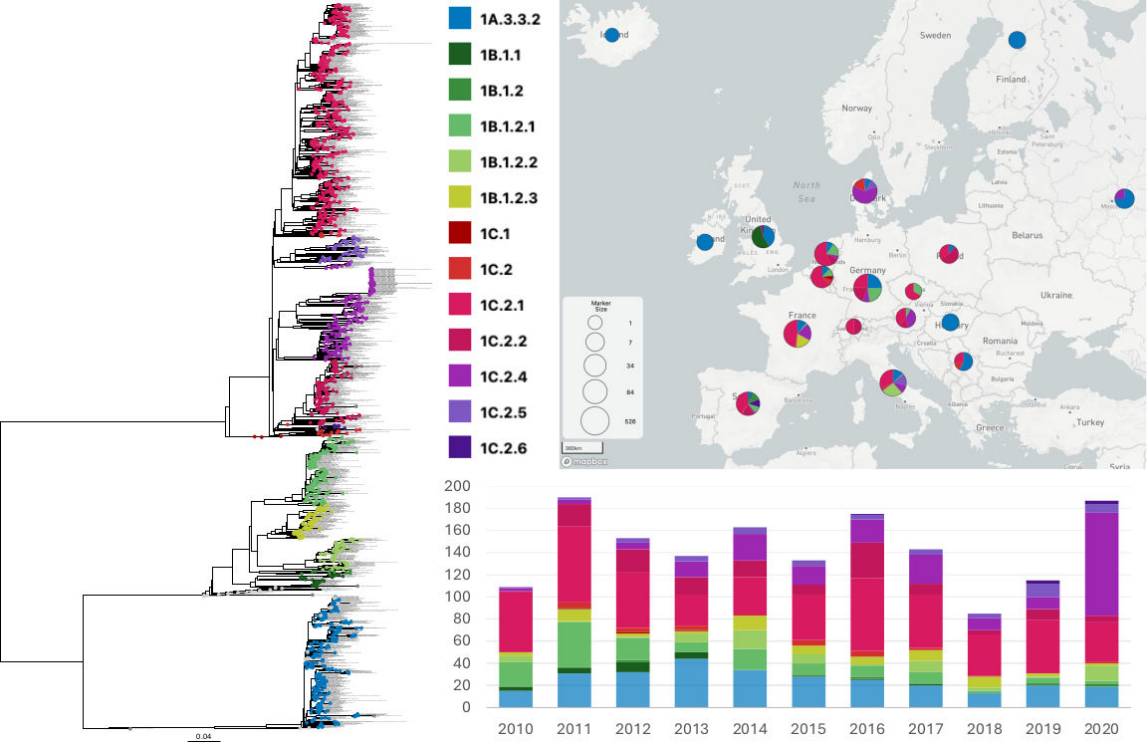
ML phylogenetic tree of influenza A H1 viruses circulating in pigs in Europe in 2010–2020. Sequence data from pigs in European countries between January 2010 until December 2020 are colored by genetic lineage as shown in the color key; strain names with identical sequences are concatenated (*n* = 1,563). Variant strains, CVVs, and human seasonal vaccine strains are included as reference strains and are colored gray (*n* = 55). The proportions of each lineage are plotted by country on a MicroReact geographical map, and the frequency of each genetic lineage detected per year is shown on the timeline (*n* = 1,697).

Geographical analysis of the circulation of swine H1 viruses inclusive from 2010 to 2020 showed different lineage distributions across time and between different countries within European pigs (Fig. 1). The co-circulation of all 1A, 1B, and 1C lineage viruses in any given year was observed between 2010 and 2020 in France, Germany, and Italy, 2011–2013 in the United Kingdom, 2011–2017 in the Netherlands, 2016–2020 in Spain, and in 2019 in Belgium. The 1C lineage viruses were the most frequently detected from 2010 to 2020. The 1A.3.3.2 (pdm09) viruses were the only clade detected in Finland, Hungary, Iceland, and the Republic of Ireland, whereas no 1A.3.3.2 viruses were detected in Austria, Czech Republic, or Switzerland. Since 2015, several larger genetic clades of 1A.3.3.2 (pdm09) viruses that have persisted in swine have been observed in Germany, Italy, and Denmark, with subsequent detections in other countries. These clades have longer branch lengths and have circulated within European pig populations for more than 1 year.

The 1B viruses in Europe arose from the introduction of human seasonal influenza virus strains with ancestral strains genetically similar to the human seasonal vaccine A/Chile/1/83. Since their first detection in Europe in 1994 (43), 1B lineage viruses have diversified into multiple clades (1B.1.1, 1B.1.2, and 1B.1.2.1, 1B.1.2.2, and 1B.1.2.3), some of which appear spatially restricted to a particular country’s swine population. The 1B.1.1 clade has only been detected in the United Kingdom except for one strain detected in Spain in 2016. The 1B.1.2 clade was only detected in Spain. The 1B.1.2.1 clade viruses have been detected in multiple countries in continental Europe and have long branch lengths suggesting undersurveillance and that these viruses are diversifying. The 1B.1.2.2 clade was only detected in Italy, and the 1B.1.2.3 clade was only detected in France.

The Eurasian avian 1C lineage has substantially diversified since their introduction into pigs to form multiple clades with complex spatial dynamics. The 1C.1 viruses were only detected in the United Kingdom. Between 2010 and 2020, the 1C.2 viruses diversified so rapidly that this clade required two new subclade designations from analysis of data within the study period, i.e., the 1C.2.4 and 1C.2.5 viruses (2). Italy, the Netherlands, Germany, Belgium, France, and Spain have multiple co-circulating 1C viruses alongside the 1C.2.1 clade. In Switzerland, only the 1C.2.1 and 1C.2.2 viruses were detected. Until 2012, the 1C.2.2 viruses were exclusively detected in Germany; however, they have since been detected in multiple other countries in Europe. Two phylogenetically similar human variants of the 1C.2.2 clade were detected in the Netherlands and Germany in 2019 and 2020, respectively, despite the relatively broad geographical and genetic diversity among other co-circulating viruses of this clade (Fig. 1). The 1C.2.4 viruses have diverged from other 1C.2.X viruses. These viruses cluster phylogenetically by country and have been detected across Europe. This clade of viruses is genetically diverse with long branch lengths relative to other 1C.2 clade viruses co-circulating in different regions. The 1C.2.5 viruses have been detected in Italy since 2010, and sporadically in Germany and Denmark, viruses cluster by country on long branch lengths. The 1C.2.6 was only detected in Spain, and this clade was only detected since 2015.

**Fig 2:**
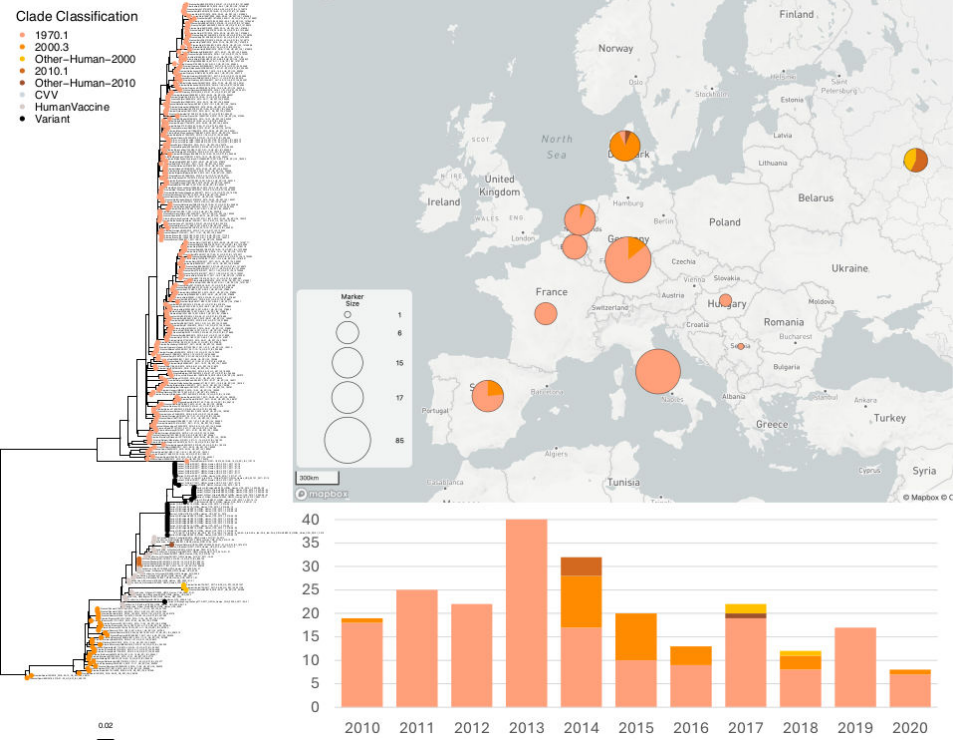
ML phylogenetic tree of influenza A H3 viruses circulating in pigs in Europe in 2010–2020. Sequence data from pigs in European countries between January 2010 until December 2020 are colored by genetic lineage as shown in the color key (*n* = 231). Variant strains, CVVs, and human seasonal vaccine strains are included as reference strains (*n* = 53). The proportions of each lineage are plotted by country on a MicroReact geographical map, and the frequency of each genetic lineage detected per year is shown on the timeline (*n* = 231).

H3 lineage viruses were less commonly detected in European pigs and were more spatially restricted than the H1 subtype viruses. Their detection frequency has decreased since 2013 (Fig. 2). H3 viruses were co-detected with H1 viruses in Denmark, the Netherlands, Belgium, France, Spain, Italy, Hungary, Serbia, and Russia but were not detected in any countries that did not also have H1 viruses. The H3 1970.1 lineage viruses have circulated in European pigs since their introduction from humans associated with the 1968 H3N2 pandemic (Fig. 2). Between 2010 and the end of 2020, the 1970.1 lineage was the predominant H3 lineage virus except in Denmark and Russia where it was not detected. Within Italy, however, genetic heterogeneity of these viruses was observed. A genetic clade of these viruses circulated in Italy between at least 2012 and 2019, which did not cluster with viruses detected elsewhere. Spain, Germany, the Netherlands, and Denmark have detected both the H3 1970.1 and a more recent lineage of H3 that emerged from a human-to-swine spillover in the early 2000s (2000.3 lineage). In Germany and the Netherlands, these 1970.1 and 2000.3 lineages co-circulated between 2014 and 2016. The 2000.3 lineage was also detected in Russia and Spain. Other unique human-to-swine spillovers without evidence for continued transmission were detected in Denmark in 2010 and in Russia in 2014 (Other-Human-2010), and a novel H3 Other-Human-2000 was detected in Russia in 2018, with sequences situated on a long branch length.

### Reassortment dynamics of surface protein-encoding genes of European swine IAVs

We generated a tanglegram to visualize HA-NA reassortment dynamics within a subsection of the collected European H1 swine IAVs (Data S3). Phylogenetic trees and respective tanglegram lines were colored by HA clade. We observed the exchange of the HA of the 1C lineage of European viruses (pink/purple) with other N1 and N2 NA segments represented by the crossing of the tanglegram lines. This provides evidence for reassortment in the HA and NA pairings of these viruses, specifically within the 1C.2.1 and 1C.2.5 clades as well as sequence data collected from Italy. In contrast, the 1A (blue), 1B (green), and 1C.2.2 viruses (dark pink) displayed sets of parallel lines that suggest preferential HA and NA pairings with limited HA and NA reassortment despite both N1 and N2 subtypes being detected. The observed reassortment of the 1C lineage HA and NA segments and pairing of the 1C HA with both N1 and N2 NAs could affect antigenic phenotype and thus the transmission dynamics of different viruses within the 1C lineage.

### Antigenic evolution in European pigs

**Fig 3:**
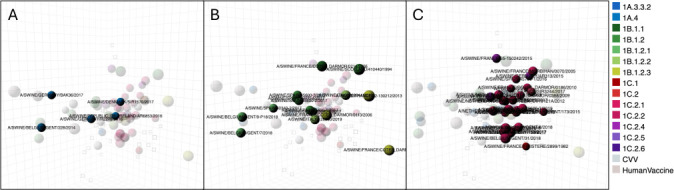
(A–C) Antigenic maps highlighting the antigenic relationships among 1A, 1B, and 1C lineage viruses in European pigs, respectively, as defined by swine antisera. Strains are represented by colored annotated spheres with the color denoting lineage as per Fig. 1. Monovalent polyclonal swine sera raised to IAVs in pigs are shown as cubes. The scale bar represents one AU or a twofold difference in HI assay titer. Panel A shows the European 1A.3.3.2 lineage viruses from swine highlighted in blue. Panel B shows the European 1B lineage viruses from swine highlighted in greens. Panel C shows the European 1C lineage viruses from swine highlighted in pink/purples. CVVs and human seasonal vaccines are shown in cream colours.

We constructed antigenic maps from data using swine sera in HI assays to characterize the antigenic properties of H1 viruses circulating in European pigs (Fig. 3A through C). Here, we observed that genetic clade did not always correspond to antigenic positioning in the map. The 1A.3.3.2 (pdm09) viruses were antigenically similar to the human seasonal pdm09 vaccine strain (A/California/4/09), but there were two outlying swine strains from Germany and Belgium (Fig. 3A). The outlying strains from Germany and Belgium were 5 AU from A/California/4/09 (Data S1).

The 1B lineage viruses showed greater antigenic variability than the 1A viruses (Fig. 3B). The earliest strains in the lineage were antigenic outliers, defined as more than 3 AU from other viruses. The H1N1 and H1N2 1B.1.2 strains isolated from pigs in Spain, which were tested, were located in the center of the antigenic map but were antigenically distinct (2–3 AU) from the tested 1B.1.2.1 strain, also from Spain. The most recent 1B.1.2.1 strains from Belgium were located as outliers on the opposite side of the map from the earliest strains we characterized and 3 AU from the 1B.1.2.1 strain from Spain. The two 1B.1.2.2 clade H1N2 strains from Italy were 3 AU apart from each other and between 1.5 and 3.4 AU from the 1B.1.2 strains from Spain. This illustrates that genotype does not always correlate with antigenic phenotype. Furthermore, within-clade antigenic diversity is a challenge for both IAV in swine vaccine strain selection and match in pigs and also for CVV strain selection and match for pre-pandemic preparedness.

The 1C viruses have circulated in European pigs since the late 1970s. They have also been associated with human variant infections in Italy, the Netherlands, Germany, France, Switzerland, and Austria. The antigenic maps revealed no clear positioning defined by the 1C clade. These viruses demonstrated significant antigenic diversity, occupying a space of 8 AU by 3 AU (Fig. 3C). The ancestral H1N1 1C.1 strain A/swine/France/Finistere-2899/1982, early clade strains from 2005 (e.g., H1N1 1C.2.1 A/swine/France/Morbihan-0070/2005), along with more recent strains from 2015 (the H1N2 1C.2.4 A/swine/France/65–150242/2015 and H1N1 1C.2.1 A/swine/Belgium/Gent-173/2015) were antigenically distinct from the other 1C antigens tested. We identified key amino acid substitutions that might be responsible for the antigenic variation that we observed in these strains. The prototype virus A/swine/France/Finistere-2899/1982 had 31 HA1 amino acid differences when compared to the 1C clade consensus sequence, with five substitutions in putative antigenic sites Ca2 (position 142), Sb (190 and 195), and Ca2 (221 and 222) (44) using H1 numbering (45). The motifs in this sequence at positions 190, 195, and 221 were not shared by any other viruses tested. There are 20 amino acid changes between the 1C consensus and A/swine/Belgium/Gent-173/2015; one change was in the putative antigenic site Cb (position 74), and the same amino acid residue was not observed in any other viruses tested, and the second was in the Sa binding site (position 155). There were seven amino acid differences between the consensus and A/swine/France/Morbihan-0070/2005. One of which was in a putative antigenic site Ca2 at position 222 with a glycine residue, as observed in A/swine/France/Finistere-2899/1982. The H1N2 virus A/swine/France/65–150242/2015 had 34 amino acid changes compared to the 1C consensus sequence, including seven of which in putative antigenic sites Cb (71), Sa (124, 125, and 155), Sb (156, 163), and Ca1 (163). Two of these residues were not observed in other sequences from viruses tested. The observed antigenic and genetic diversity makes selection of one strain to cover all current 1C antigenic diversity unlikely.

### Antigenic characterization of European swine viruses by ferret antisera

**Fig 4:**
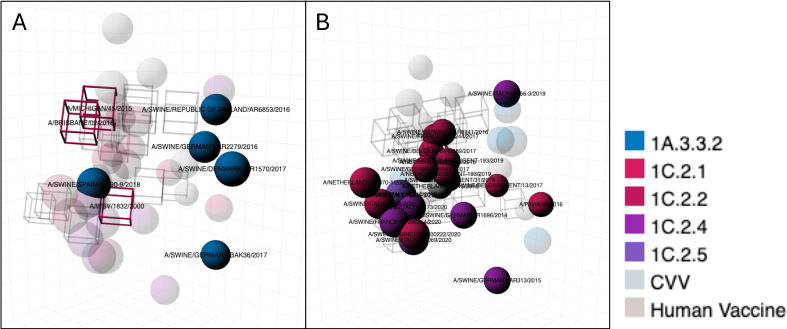
(A, B) Antigenic map highlighting the antigenic relationships among the swine European 1A and 1C lineages, respectively, as defined by ferret antisera. Strains are represented by colored spheres with the color denoting lineage as per Fig. 1. Polyclonal ferret sera raised to swine IAVs are shown as cubes. The scale bar represents one AU or a twofold difference in HI assay titer. Panel A shows the European 1A.3.3.2 clade viruses from swine highlighted in blue. Panel B shows the European 1C lineage viruses from swine highlighted in pinks/purples. CVVs and human seasonal vaccines are shown in cream colors.

We constructed antigenic maps from data using ferret sera raised against swine vaccine-representative strains, CVVs, and human seasonal vaccines in HI assays to characterize the antigenic properties of 1A and 1C European swine viruses as this is relevant for assessment of CVVs for pre-pandemic preparedness. No human variant cases of 1B viruses had been observed in Europe up to the end of 2020. We observed that within the 1A lineage (Fig. 4A), an antigenically similar group of viruses was 2–4 AU distance to a recent 1A.3.3.2 (pdm09) human seasonal vaccine strain, A/Brisbane/02/2018. A swine strain from Germany from the 1A.3.3.2 clade was an antigenic outlier at more than 7 AU from the human seasonal A/Brisbane/02/2018.

The 1C lineage viruses (Fig. 4B) had more defined positioning in ferrets compared to swine, and we observed two separate antigenic clusters as shown on the 3D antigenic map. One cluster was comprised of European swine strains that circulated between 2017 and 2019, which were antigenically similar to the 1C.2.1 CVV strain A/Netherlands/3315/2016 and the 1C.2.2 human variant strain A/Netherlands/Gent-193/2019. The other main cluster contained swine viruses from 2019 to 2020 that were antigenically similar to the 1C.2.1 CVV A/Netherlands/10370-1/2020. There were outlier swine strains collected in Italy (clade 1C.2.4), Germany (clade 1C.2.4), and Belgium (clade 1C.2.1) that were located around the antigenic map. The isolates from Italy and Belgium were located at 3 AU from the main clusters, while A/swine/Germany/AR313/2015 was located at 4 and 6 AU from the two main antigenic clusters.

### Comparison of immune response characterized using swine or ferret antisera

**Fig 5:**
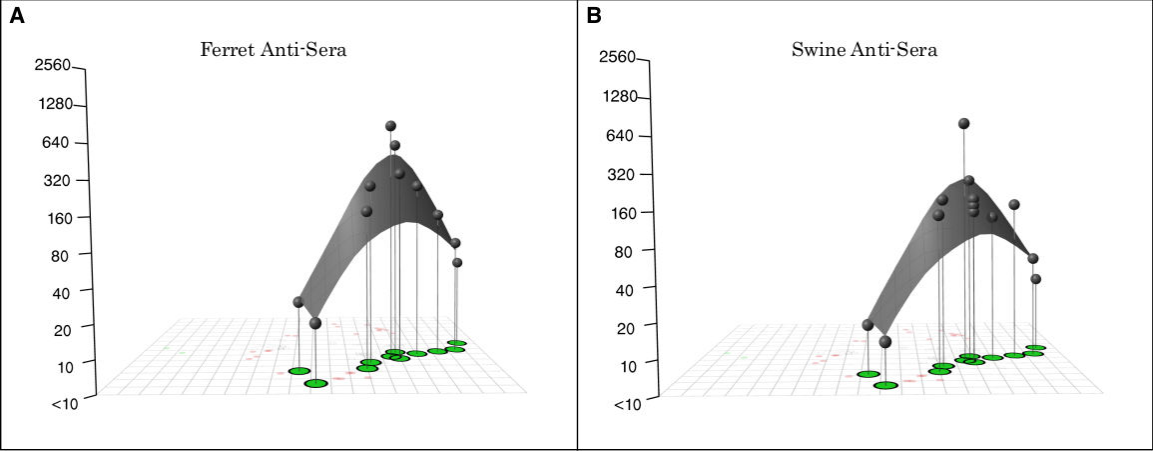
(A, B) Antibody landscaping representing the immunological response to a range of European H1 strains in post-infection ferret antisera and post-vaccination polyclonal swine antisera. The *X* and *Y* axes represent the coordinates of the virus strains derived from the 2D antigenic maps. The European H1 strains have been colored green. The *Z* axis represents the HI GMT at each antigenic point for each host population. Panel A shows the results using the 2D ferret antigenic map and post-infection ferret antisera, and panel B shows the results using the 2D swine antigenic map and post-vaccination polyclonal swine antisera.

Swine antisera raised against IAVs from pigs are routinely used in HI assays to characterize within-host influenza evolution. However, vaccine strain assessments for humans are commonly undertaken using polyclonal ferret antisera raised to vaccine strains or CVVs for human pre-pandemic risk assessment. Here, we compared the antigenic relationships among a subset of strains using both swine and ferret polyclonal antisera to carry out antibody landscaping (Fig. 5). Our focus was the serological response of these hosts to IAV, whether through vaccination (swine) or infection (ferret), and we interpolated the surfaces over the antigenic map positioned with ferret antisera.

There are two measures that can be analysed from antibody landscapes: the magnitude (level of antibody titer and height of the landscape) and breadth (span of the smooth surface) of the antibody response. The magnitude and breadth of the antibody response were very similar in both animal models, demonstrating the same level of cross-reaction across the European H1 strains whether detected by ferret (Fig. 5A) or swine (Fig. 5B) sera. There was no statistical difference between the antibody response of the two animal models after immunization with European H1 strains.

### Vaccination in pigs

**Fig 6:**
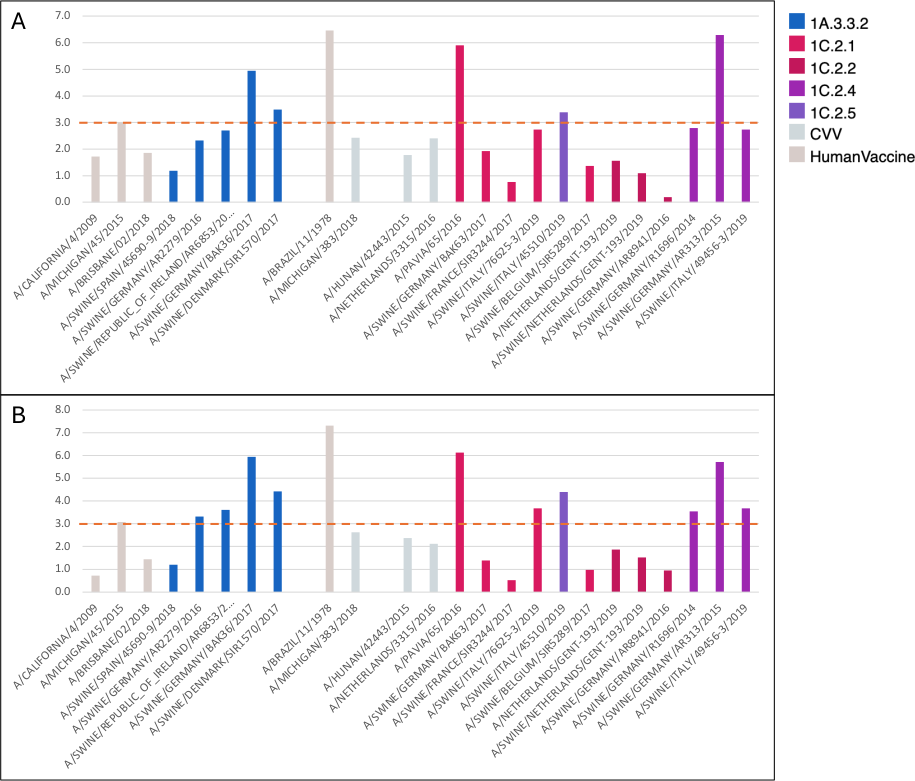
(A, B) Antigenic distances between H1 test viruses and European swine influenza vaccine-representative strains. Test viruses are colored by lineage, CVV or vaccine seed strain. Panel A against the 1A.3.3.2 clade A/Jena/Msv-Vi5258/2009, and panel B against the 1C.2.2 clade A/Swine/Haselunne/Idt2617/2003 vaccine-representative strains. The height of the bar represents the AUs between the test virus and the vaccine-representative strain. Three AU differences between vaccine sera and test antigen are considered antigenically different enough to trigger an update to the human influenza vaccine (38).

Ferret-derived sera are sometimes raised against swine vaccine-representative strains in Europe to determine the likely antigenic vaccine match to contemporary viruses. Figure 6 shows the antigenic distances from the 1A and 1C strains to the ferret-derived sera raised against vaccine-representative strains used in European pigs from European isolates collected between 2014 and the end of 2020, human seasonal strains, and CVVs. We observed that clade 1A.3.3.2 H1N2 strains from Germany and Denmark were over 3 AU and up to 5 AU away from the 1A.3.3.2 swine vaccine-representative strain A/Jena/Msv-Vi5258/2009 (Fig. 6A). 1C viruses also showed heterogeneity in cross-reactivity to the 1C.2.2 ferret serum raised against the vaccine-representative strain A/Swine/Haselunne/Idt2617/2003. Four of eight H1N1 and one of two H1N2 1C.2.1 clade strains were over 3 AU from this vaccine-representative strain. One H1N1 clade 1C.2.2 strain was over 3 AU from the within-clade vaccine-representative strain. All 1C.2.4 strains were between 3 and 6 AU from the vaccine-representative strain, and the H1N2 1C.2.5 was 2.7 AU from the vaccine-representative strain (Fig. 6B) (Data S2).

### Human serology

**Fig 7:**
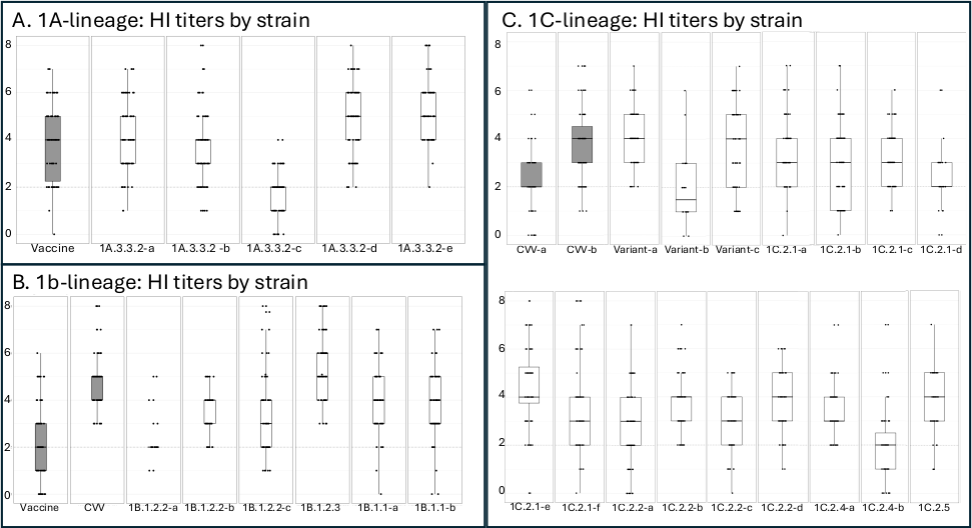
(A–C) Log_2_ of GMT of HI responses of human antisera against circulating European H1 swine viruses. Boxplots depict the 25% (Q1) and 75% (Q3) quantile. The whiskers illustrate the variability outside the Q1 and Q3. Points positioned outside of the whiskers of the boxplot are outliers. Each dot represents the GMT log_2_ (HI titer/10) of each human antiserum against each virus strain. The dotted line indicates the positive HI titer threshold (1:40 or 2 GMT) purported to confer protection against infection. The boxplots for the human vaccine strains or CVVs have been colored in grey. Panel A shows sera tested against 1A human vaccine virus A/Brisbane/02/18 and European Swine 1A.3.3.2 viruses: (A) A/Swine/Germany/R2279/2016, (**B**) A/Swine/Republic Of Ireland/Ar6853/2016, (**C**) A/Swine/Germany/Bak36/2017, (**D**) A/Swine/Denmark/Sir1570/2017, and (E) A/swine/Italy/241572/2020. Panel B shows sera tested against human vaccine virus A/Brazil/1/78, CVV A/Michigan/383/18, 1B.1.2.2 viruses: (A) A/Swine/Italy/126300/2019, (**B**) A/Swine/Italy/185280/2020, and (C) A/Swine/Italy/202244/2019, 1B.1.2.3 A/Swine/France/29–200240/2020 and 1B.1.1 strains: (a) A/Swine/England/208046/2018 and (b) A/Swine/England/062058/2018. Panel C shows sera tested against the 1C.2.3 and 1C.2.1 CVVs: (A) A/Hunan/42443/15 and (B) A/Netherlands/3315/2016, respectively, which are colored grey. Variant strains the 1C.2.2: (A) A/Netherlands/Gent-193/2019 the 1C.2.1, (B) A/Pavia/65/2016, and (C) A/Netherlands/Gent-193/2019. The 1C.2.1 strains: (A) A/swine/Belgium/SIR5289/2017, (**B**) A/swine/France/SIR3244/2017, (C) A/swine/Germany/BAK63/2017, (**D**) A/swine/Italy/6625-3/2019, (**E**) A/swine/France/85–200222/2020, and (**F**) A/swine/Spain/45690-4/2018. The 1C.2.2 isolates: (A) A/swine/Germany/AR8941/2016, (**B**) A/swine/Netherlands/Gent-193/2019, (C) A/swine/Spain/6370-1/2018, and (D) A/swine/Spain/6370-2/2018. The 1C.2.4 isolates: (A) A/swine/Germany/R1696/2014 and (B) A/swine/Germany/AR313/2015. Finally, the 1C.2.5 A/swine/Italy/45510/2019.

Based on our phylogenetic analysis of the European swine H1 lineages, we selected strains to test in HI assays using two human antisera cohorts (post-vaccination and post-infection) to characterize potential immunity to swine strains and assess the relative risk of incursion into humans (Fig. 7). We also tested the cohort antibody response to human vaccine strains (boxplots colored in grey) to determine if any current seasonal vaccine strains would offer cross-neutralization against swine strains and to ensure that the cohorts included “low,” “medium,” and “high” responders to the seasonal vaccine (Fig. 7).

We selected the A/Brisbane/02/2018 (1A.3.3.2) human vaccine strain as a human seasonal representative virus to be tested against human cohort sera. An HI titer of 1:40 or a GMT of 2 is generally considered the putative threshold that would confer protection against a future influenza infection. We plotted the HI titers obtained against the reference human seasonal vaccine and the representative swine influenza strains (Fig. 7) to characterize the distribution of population immunity.

For each of the H1N1 human seasonal vaccine strain A/Brisbane/02/2018 and 1A.3.3.2 swine strains A/swine/Germany/R2279/2016 and A/swine/Republic of Ireland/AR6853/2016 (which were less than 3 AUs from the representative putative introduction strain and CVV A/California/4/09 [Data S2]), we observed that the majority of the cohort had titers greater than 1:40 and a median response of 4 GMT. Two additional swine 1A.3.3.2 strains, an H1N2 A/swine/Denmark/SIR1570/2017 and H1N1 A/swine/Italy/241572/2020, showed a similar distribution of titers, all of which had titers greater than 1:40 and a median of 5 GMT for both strains. The final 1A.3.3.2 H1N2 strain A/swine/Germany/BAK36/2017 (which was 5.8 AU from the CVV [Data S2]) was poorly recognized by the majority of the cohort antisera, the median was below 1:40, and some antisera showed no cross-reactivity. Individuals who had titers of less than 1:40 were distributed across all decades of birth included in the study.

For the human cohort immunity assessment of the human seasonal 1B lineage, we selected two vaccine strains: A/Brazil/1/1978, the closest human seasonal strain, which is a putative ancestor to the introduction of the 1B lineage into swine, and A/Michigan/383/2018 (1B.2.1), which was the only available CVV from the 1B lineage. The distribution of titers against A/Brazil/1/1978 was spread widely, with a median of two GMT, though some individuals had no cross-reactivity to this strain. Sixteen of the 26 low reactors were born after 1979, and seven had no age information available. All individuals had titers above 3 GMT to the 1B.2.1 CVV A/Michigan/383/2018. All 1B test viruses gave median titers above 2 GMT (range 0–8 GMT), though two of each of the H1N2 1B.1.2.2 and 1B.1.1 viruses were poorly recognised by some individuals.

The 1C lineage viruses which circulate in pigs have caused variant infections in humans in a number of European countries and in China. To analyze the cross-reactivity of human sera to Eurasian avian-like 1C lineage viruses, we selected CVVs A/Hunan/42443/2015 (1C.2.3) and A/Netherlands/3315/2016 (1C.2.1). We also selected the three variant strains A/Netherlands/Gent-193/2019 (1C.2.2), A/Pavia/65/2016 (1C.2.1), and A/Netherlands/10370/2020 (1C.2.1) from which a CVV has been proposed. Given the demonstrated antigenic heterogeneity and genetic diversity of the 1C lineage viruses, we selected 12 swine 1C viruses isolated from various European countries and from various clades. This includes 1C.2.1 strains (*n* = 7) from France, Germany, Spain, Italy, and Belgium; 1C.2.2 strains (*n* = 3) from Germany, Spain, and the Netherlands; a 1C.2.4 strain from Germany; and a 1C.2.5 strain from Italy.

The median antibody response against most 1C lineage European viruses, CVVs, and variants was over 2 GMT, and most European viruses had greater recognition from the cohort sera than the 1C.2.3 CVV A/Hunan/42443/2015, including the 1C.2.1 CVV A/Netherlands/3315/2016. The exception was A/Pavia/65/2016, an H1N1 1C.2.1 antigenic outlier (Fig. 4B), which had a median GMT of 1.5 with GMT ranging from 0 to 6. For three H1N1 viruses tested (1C.2.2s A/Netherlands/Gent-193/2019, A/swine/Netherlands/Gent-193/2019, and the 1C.2.4 A/Swine/Germany/R1696/2014), the entire cohort had a GMT of greater than 2, and interestingly, these three viruses were positioned more centrally in the antigenic map (Fig. 4B). For the other nine viruses tested, there was heterogeneity in cohort titer, and a proportion of individuals had titers of less than 2 GMT, suggesting a variable infection risk among the cohort.

## DISCUSSION

We found that multiple HA lineages are co-detected in various European countries, in combination with different NAs and at different times. Genetic diversity has increased throughout the study timeframe, resulting in the need to update nomenclature to include newly detected and expanding lineages, such as the 1C.2.5 clades. Of the 1A lineage viruses, only the 1A.3.3.2 viruses have been maintained in Europe, and the previously circulating classical-like 1A viruses have not been detected since the early 1990s. Interestingly, introductions of human seasonal IAV into swine did not always result in onward transmission for more than a year, contrasting data from the United States and Brazil (46, 47). 1B and 1C lineage viruses have complex spatial dynamics which may be a result of different pig sub-populations and production systems across the European continent. The H3 viruses that have been detected in Europe are diverse and are a result of multiple reverse zoonoses events with maintained circulation (1, 10). Long phylogenetic branch lengths indicate undersurveilled evolution, suggesting the maintenance of these lineages in pig populations. Many of the European H3 viruses have not, to our knowledge, been comprehensively antigenically characterized against putative human seasonal ancestors, CVVs, and human sera to understand their potential pandemic risk. Furthermore, surveillance programs vary between European countries, with varied sampling frequencies, caused by the varied production systems through the countries and the fact that IAV is not a notifiable disease.

Such genetic diversity has led to a complex antigenic profile in pigs, where some viruses have not substantially evolved phenotypically from their putative human seasonal ancestors, whereas others within the same lineage in other countries or even within the same country have markedly drifted. Despite the observations presented here, we still do not have a complete picture of the true diversity as many pig populations in Europe remain poorly sampled, or data are not publicly available. Some production units have substantial longitudinal problems with influenza, whereas others detect disease infrequently. This is supported by findings of reassortment of IAV in swine clades indicating not only co-detection but co-circulation (1, 3, 4, 9–11). The underlying factors contributing to this complex epidemiology are poorly understood. However, this onward and divergent evolution in different swine populations reflects a challenge for control in pigs. For example, one pdm09 antigen may not be sufficient to cover observed antigenic diversity of the 1A.3.3.2 clade viruses in Europe, and antigens should be appropriately tailored to viruses which are known to be circulating in that region. Furthermore, in humans, some of these 1A.3.3.2 viruses are likely antigenically drifted from immunity which would be derived from vaccination or natural exposure to the pdm09 in human populations, meaning these groups of viruses pose an increased pandemic risk.

IAV in swine has consistently posed a challenge to animal production by contributing to swine respiratory disease complex and reducing productivity. Although vaccination is used in some European countries to attempt to control disease, it is not used in all, and in some EU Member States or production systems, there has been evidence of loss of vaccine efficacy (Deblanc et al., 2020 [48]). Factors which influence vaccine efficacy include the age of the animal and stage of the production cycle, the potential confounding effect of maternally derived antibody, and less than optimal strain match between circulating and vaccine strains (8). We showed that the heterogeneity of circulating virus in European pigs is significant at the geographical, genetic, and antigenic levels. We reveal antigenic drift from vaccine-representative strains (greater than 3 AU) for H1N2 viruses of the 1A.3.3.2 clade: some H1N1 viruses of the 1C2.1 and H1N1 and N2 1C.2.4 and H1N2 1C.2.5 clade viruses. Such large antigenic distances are likely to impact vaccine efficacy in the field, as reported by Deblanc et al. (48) and Richard et al. (49) on the emergence of the H1N2 clade 1C.2.4 virus in France, despite adjuvant use to broaden a response (37). Subsequent work should focus on defining correlates of cross-protection for the observed antigenic diversity in pigs to provide a quantitative basis for when vaccines might need updating. Furthermore, the impact of using poorly matched vaccines upon future virus evolution is unknown at the population level and requires evaluation. As though vaccination protects against disease, it may not reduce shedding, nor transmission between nor within herds. This may contribute to virus selection and thus change the rate of antigenic evolution.

Phenotypic evolutionary assessment of influenza viruses in pigs has commonly focused on using swine antisera in HI assays. This is entirely logical as the pig serves as a natural host model for the evolution of the viruses within pig populations. However, assessment of influenza in humans commonly relies on responses to influenza viruses generated by ferrets, as does some vaccine strain selection in other animals (e.g., horses). To our knowledge, this is the first quantitative and comparative assessment of the two hosts’ immunological responses to influenza and whether one model or the other (pig or ferret) is more appropriate to assess phenotypic evolution in different scenarios. Here, we showed, despite slightly varied immunization regimes, with matched viruses and antisera raised in both hosts, that the breadth and relative magnitude of the immunological response was equivalent using antibody landscaping. This is critical as it allows triage of viruses circulating in pigs to be validly undertaken using either model system and extrapolation between the two in selecting viruses of interest for further assessment such as antigenic variance from vaccine strain or for pandemic preparedness. This also indicates that either vaccination (in pigs) or infection (in ferrets) as the antigen presentation route is equivalent, demonstrating a level of tolerance between different immunization regimes, for example, when comparing two doses of vaccine in pigs and one dose of live virus in ferrets.

Since 2017, there have been several instances of variant cases (human infections with swine IAVs) in Europe. These human variant cases have not been limited to a particular swine HA lineage or country (WHO, 2020; WHO, 2021a; WHO 2021b; 18–20). Rather, they have been sporadically detected primarily through influenza-like illness and subsequently reported according to international health regulation requirements. We analyzed representatives of the sampled diversity in European pigs along with reference human seasonal or CVVs to quantify the immunological profile of two human cohorts to these strains and assess the potential for these viruses to infect humans and spread. Vandoorn et al. (50) found age- and lineage-dependent cross-reactive human antibodies against swine strains tested in their study. In our study, as with the antigenic analyses with swine and ferret antisera, HA lineage did not reliably predict immunological profile with human sera. Within lineages, there were some strains that could be considered “riskier” as there was poor immunological recognition by human cohorts, particularly for some of the tested 1A.3.3.2 (pdm09) and 1C lineage strains, especially those viruses which were antigenically distant from human seasonal vaccines or CVVs.

There are likely other factors that contribute to interspecies transmission and subsequent spread in human populations—this includes the risk interface, immunological status of the host based on prior exposure and vaccination, and virus factors such as HA-NA combinations and transmission efficiency. In some cases, there may be a “putative protective” effect from a human seasonal N1 rather than an N2 combination that was detected in swine viruses that had antigenically drifted within the HA in swine. In general, prior studies have demonstrated significantly less antigenic diversity in swine NA than in the HA gene (6, 51), and extending pandemic preparedness strategies to consider the NAs may overcome the challenge represented by significant diversity in the HA gene in swine in Europe and globally. This effect may not be revealed by assessment with the HI assay, and as such, human cohort serological analyses should be incorporated into a formal pandemic preparedness risk-assessment pipeline with further downstream steps to assess virus neutralization, human receptor binding, and other potential risk factors such as anti-viral resistance.

## Data Availability

The data associated with this study are available as supplemental material and are posted at https://github.com/acoggon3/Euro-Swine-Risk/tree/main/Accessions.

## SUPPLEMENTAL MATERIAL

Data S1 jvi.00306-25-s0001.xlsx Swine antisera and test viruses used in this study, including subtype and clade information where available.

Data S2 jvi.00306-25-s0002.xlsx Ferret antisera and test viruses used in this study, including subtype and clade information.

Data S3 jvi.00306-25-s0003.pdf H1-NA tanglegram.

Data S4 jvi.00306-25-s0004.html Antigenic map for swine H1.

Data S5 jvi.00306-25-s0005.html Antigenic map for swine H1 viruses against ferret antisera.

Data S6 jvi.00306-25-s0006.xlsx Amino acid differences between the consensus sequence of 1C viruses and each test virus used in this study.

Legend S4 jvi.00306-25-s0007.docx Legend for Data S4.

Legend S5 jvi.00306-25-s0008.docx Legend for Data S5.

ASM does not own the copyrights to Supplemental Material that may be linked to, or accessed through, an article. The authors have granted ASM a non-exclusive, world-wide license to publish the Supplemental Material files. Please contact the corresponding author directly for reuse.
