# Supplementary material for: Quantifying the zoonotic risk profile of European influenza A viruses in swine from 2010 to 2020 inclusive
Source: J Virol. 2025 Jun 4;99(7):e00306-25. doi: 10.1128/jvi.00306-25 (PMC12288490; doi:10.1128/jvi.00306-25)
Supplement: Data S3 — H1-NA tanglegram. [file jvi.00306-25-s0003.pdf]

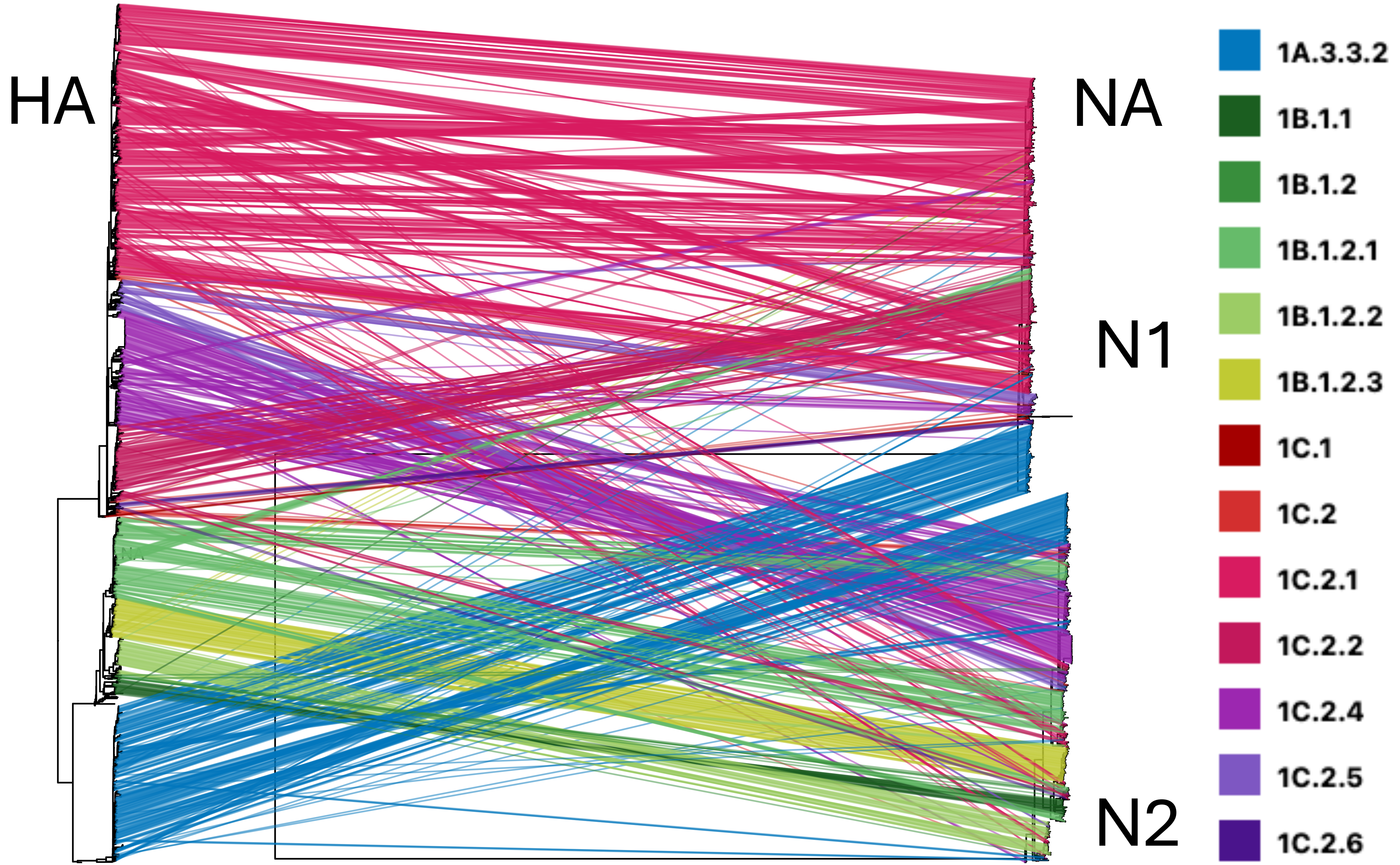

Supplementary data 3: Maximum likelihood phylogenetic trees of HA and NA segment pairings of H1 viruses circulating in European pigs where sequence data were available for both segments (n=1424). Identical tips are connected with lines coloured according to HA genetic lineage as per figure 1 in order to visualise the diversity of HA and NA gene segment pairings among H1 European viruses. Parallel lines indicate genetically stable pairings and crossed lines indicate heterogeneity in gene pairings.
