# Supplementary material for: Quantifying the zoonotic risk profile of European influenza A viruses in swine from 2010 to 2020 inclusive
Source: J Virol. 2025 Jun 4;99(7):e00306-25. doi: 10.1128/jvi.00306-25 (PMC12288490; doi:10.1128/jvi.00306-25)
Supplement: Data S4 — Antigenic map for swine H1. [file jvi.00306-25-s0004.html]

RacViewer
