# Supplementary material for: Quantifying the zoonotic risk profile of European influenza A viruses in swine from 2010 to 2020 inclusive
Source: J Virol. 2025 Jun 4;99(7):e00306-25. doi: 10.1128/jvi.00306-25 (PMC12288490; doi:10.1128/jvi.00306-25)
Supplement: Legend S4 — Legend for Data S4. [file jvi.00306-25-s0007.docx]

Supplementary data 4: Additional data for figure 3 - an interactive HTML file of the Antigenic maps highlighting the antigenic relationships among 1A, 1B and 1C lineage viruses in European pigs respectively as defined by swine anti-sera. Strains are represented by coloured annotated spheres with the colour denoting lineage as per Figure 1. Monovalent polyclonal swine sera raised to influenza A viruses in pigs are shown as cubes. The scale bar represents one antigenic unit (AU) or a two-fold difference in HI assay titre. European 1A.3.3.2 lineage viruses from swine are highlighted in blue, 1B lineage viruses in greens and 1C lineage viruses in pink/purples. CVVs and human seasonal vaccines are shown in cream colours.
